# Heterologous Ad26/Ad5 adenovirus-vectored vaccines elicited SARS-CoV-2-specific antibody responses with potent Fc activities

**DOI:** 10.3389/fimmu.2024.1382619

**Published:** 2024-05-08

**Authors:** Jéromine Klingler, Shreyas Kowdle, Juan C. Bandres, Rozita Emami-Gorizi, Raymond A. Alvarez, Priyanka G. Rao, Fatima Amanat, Charles Gleason, Giulio Kleiner, Viviana Simon, Alexis Edelstein, Claudia Perandones, Chitra Upadhyay, Benhur Lee, Catarina E. Hioe

**Affiliations:** ^1^ Division of Infectious Diseases, Department of Medicine, Icahn School of Medicine at Mount Sinai, New York, NY, United States; ^2^ James J. Peters VA Medical Center, Bronx, NY, United States; ^3^ Department of Microbiology, Icahn School of Medicine at Mount Sinai, New York, NY, United States; ^4^ Graduate School of Biomedical Sciences, Icahn School of Medicine at Mount Sinai, New York, NY, United States; ^5^ Center for Vaccine Research and Pandemic Preparedness, Icahn School of Medicine at Mount Sinai, New York, NY, United States; ^6^ Department of Pathology, Molecular and Cell Based Medicine, Icahn School of Medicine at Mount Sinai, New York, NY, United States; ^7^ Global Health and Emerging Pathogens Institute, Icahn School of Medicine at Mount Sinai, New York, NY, United States; ^8^ Administración Nacional de Laboratorios e Institutos de Salud (ANLIS) Dr. Carlos G. Malbrán, Buenos Aires, Argentina

**Keywords:** COVID-19, vaccines, adaptive immunity, immunoglobulins, antibodies, Fc functions, SARS-CoV-2

## Abstract

**Introduction:**

Antibodies against the SARS-CoV-2 spike protein are a critical immune determinant for protection against the virus. While virus neutralization is a key function of spike-specific antibodies, antibodies also mediate Fc-dependent activities that can play a role in protection or pathogenesis.

**Methods:**

This study characterized serum antibody responses elicited after two doses of heterologous adenovirus-vectored (Ad26/ Ad5) vaccines.

**Results:**

Vaccine-induced antibody binding titers and Fc-mediated functions decreased over six months, while neutralization titers remained stable. Comparison of antibody isotypes elicited after Ad26/Ad5 vs. LNP-mRNA vaccination and after infection showed that anti-spike IgG1 were dominant and produced to high levels in all groups. The Ad26/Ad5 vaccines also induced IgG4 but not IgG2 and IgG3, whereas the LNP-mRNA vaccines elicited a full Ig spectrum (IgM, IgG1-4, IgA1-2). Convalescent COVID-19 patients had mainly IgM and IgA1 alongside IgG1. Despite these differences, the neutralization potencies against early variants were similar. However, both vaccine groups had antibodies with greater Fc potencies of binding complement and Fcg receptors than the COVID-19 group. The Ad26/Ad5 group also displayed a greater potency of RBD-specific antibody-mediated cellular phagocytosis.

**Discussion:**

Antibodies with distinctive quality were induced by different vaccines and infection. The data imply the utility of different vaccine platforms to elicit antibody responses with fine-tuned Fc activities.

## Introduction

The emergence of the coronavirus disease (COVID-19) has led to an unprecedented global pandemic, causing almost seven million deaths worldwide. Many vaccines for the severe acute respiratory syndrome coronavirus 2 virus (SARS-CoV-2) have been developed to combat this pandemic, and most are based on the SARS-CoV-2 spike protein as the key vaccine component designed to elicit virus neutralizing antibodies (1). Indeed, the spike protein is the main target of neutralizing antibodies which block virus binding to the angiotensin-converting enzyme 2 (ACE2) receptor. However, through their Fc fragments, antibodies also can engage the innate immune system to mediate effector functions, such as complement-mediated lysis and antibody-dependent cellular cytotoxicity and phagocytosis (ADCC and ADCP), by triggering the complement cascade or activating natural killer cells, neutrophils, macrophages, and other innate immune cells (2).

One of the COVID-19 vaccines that were deployed early during the pandemic and approved for clinical use in nearly 70 nations including Russia, Argentina, Brazil, Hungary, India and the Philippines is the Sputnik V or Gam-COVID-Vac vaccine (3). The Sputnik V vaccination regimen comprises of recombinant heterologous adenovirus types 26 and 5 vectors expressing the Wuhan Hu-1 spike protein (Ad26 and Ad5), with the Ad26 vaccine administered as the first dose and the Ad5 vaccine as the second dose. The Ad26/Ad5 vaccines demonstrated a 91.6% efficacy rate at preventing symptomatic COVID-19 infection and were 100% effective at preventing severe infection during the dominance of early SARS-CoV-2 variants (4–8). However, the neutralization efficacy against later SARS-CoV-2 variants like B.1.351 (Beta), P.1 (Gamma), B.1.617, and B.A1 (Omicron) was shown to be lower than that against the Wuhan-Hu-1 G614-variant (D614G) (9–16), similar to the decreased neutralization titers reported for the LNP-mRNA vaccines (Pfizer/BioNtech BNT162b2 and Moderna mRNA-1273) and for the other vectored vaccine platforms such as Janssen Ad26.COV2.S and AstraZeneca ChAdOx1 (17–19).

Evaluation of the vaccine efficacy has been based mainly on the neutralization titers of vaccine-induced antibodies, although the non-neutralizing Fc-mediated activity of these antibodies also have been implicated in protection (20, 21). Indeed, despite a reduction in neutralization capacities, the Ad26/Ad5 vaccine remained effective against hospitalization and severe lung injury associated with SARS-CoV-2 Omicron (22), and this could be explained by the protective activities of cellular immunity and/or antibody Fc-mediated activities. Importantly, past studies showed that antibody Fc functions such as antibody-dependent monocyte-mediated phagocytosis (ADMP) were detected up to 6 months post-vaccination with Sputnik V and 8 months post-infection (23, 24). However, studies evaluating the Fc activities of antibodies elicited by the Ad26/Ad5 vaccines as compared to other vaccines or natural infection have been scant.

Here, we conducted a longitudinal analysis of serum antibody responses elicited by the Ad26/Ad5 vaccines. We measured antibody binding levels, Ig isotypes, neutralizing titers, and Fc-mediated functions for up to six months after the second vaccine dose. We also compared the Ig isotypes and functions of antibodies elicited by Ad26/Ad5 vaccines, LNP-mRNA vaccines, and after infection.

## Results

### Serum antibody responses to SARS-CoV-2 detected over time after Ad26/Ad5 vaccination

**Figure 1:**
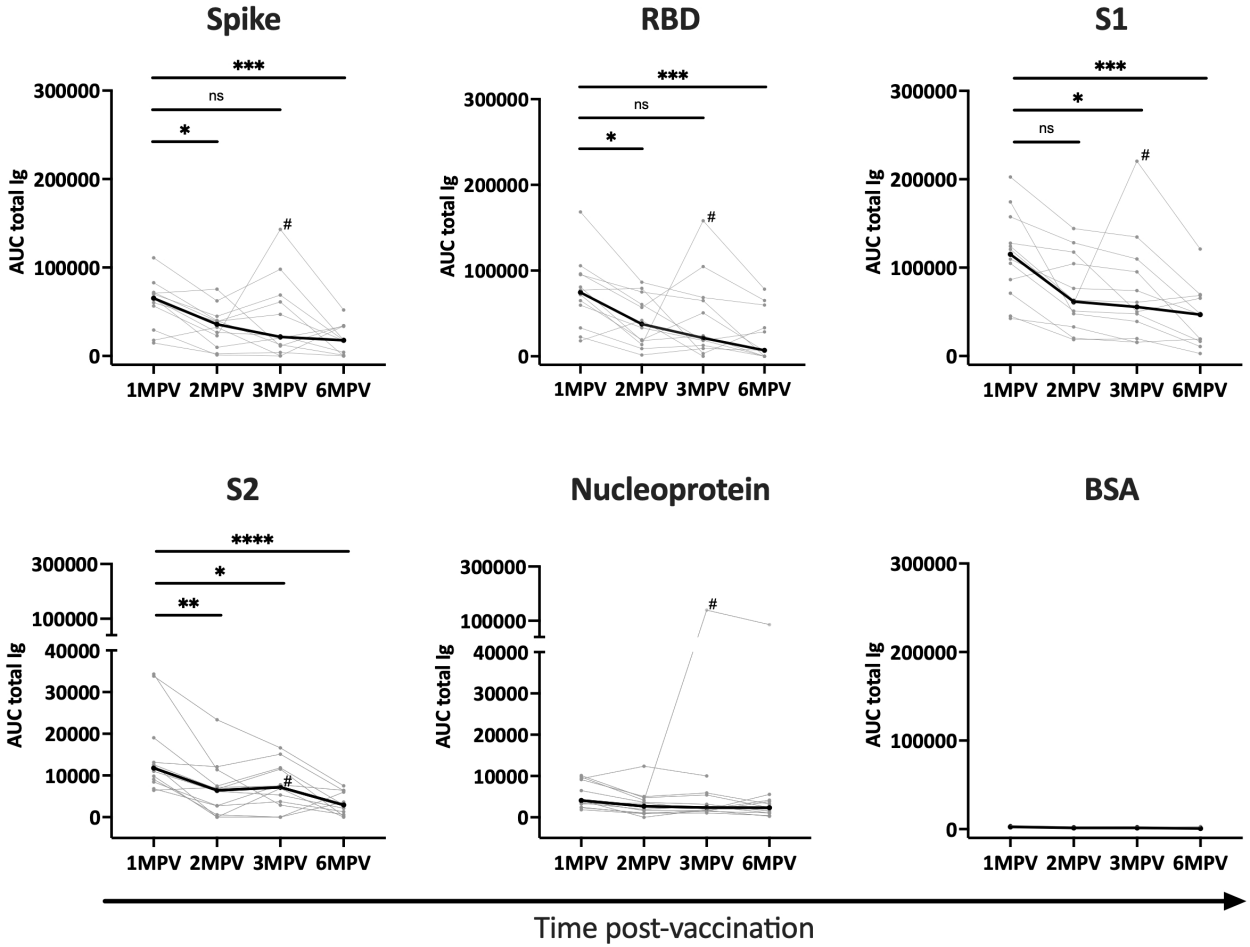
Antibody responses to SARS-CoV-2 detected over time after Ad26/Ad5 vaccination. Longitudinal total Ig responses against SARS-CoV-2 spike, RBD, S1, S2, and nucleoprotein antigens were measured in sera from 12 Ad26/Ad5-vaccinated individuals collected at 1-, 2-, 3- and 6-months post-vaccination (MPV). BSA was tested as a negative control. The secondary anti-Ig antibodies used in this multiplex bead assay detected all major serum Ig isotypes (IgG, IgM, IgA). AUC, area under the curve. The thin lines show data of individual vaccinees, the thicker black lines represent the median. # denotes subject 6 who were infected with SARS-CoV-2 prior to 3 MPV. ****, p<0.0001; ***, p<0.001; **, p<0.01; *, p<0.05; ns, p ≥0.05 by Friedman test followed by Dunn’s multiple comparisons test.

Serum specimens from 12 healthy recipients of COVID-19 Ad26/Ad5 vaccines were collected at 1-, 2-, 3-, and 6-month(s) post-second vaccine dose (MPV). The samples were titrated to measure total Ig antibody responses to SARS-CoV-2 spike, RBD, S1, S2, nucleoprotein antigens and bovine serum albumin (BSA) as a negative control (**Supplementary Figure S1**). Areas under the curve (AUC) were calculated and plotted to examine changes in antibody levels over time (**Figure 1**). Spike-, RBD-, and S1- specific responses were detectable in all 12 vaccinees at 1 MPV and, as expected, declined over time. From 1 MPV to 6 MPV, the median values significantly decreased by 2.4 to 5.3-fold for the three different antigens (**Figure 1**), consistent with previous observations (15). The anti-S2 responses were detected only in a fraction of vaccinees at low levels (**Supplementary Figure S1**) and also dropped over time (median 3.6-fold decrease from 1 MPV to 6 MPV) (**Figure 1**). The responses to nucleoprotein were below or close to the BSA control and did not change over time, except for one individual (subject 6) who demonstrated elevated levels of anti-nucleoprotein antibodies at 3 MPV and 6 MPV (**Figure 1**, **Supplementary Figure S1**), indicating a breakthrough infection prior to the 3 MPV time point.

### Serum antibody isotypes against SARS-CoV-2 elicited at the different time points after Ad26/Ad5 vaccination

**Figure 2:**
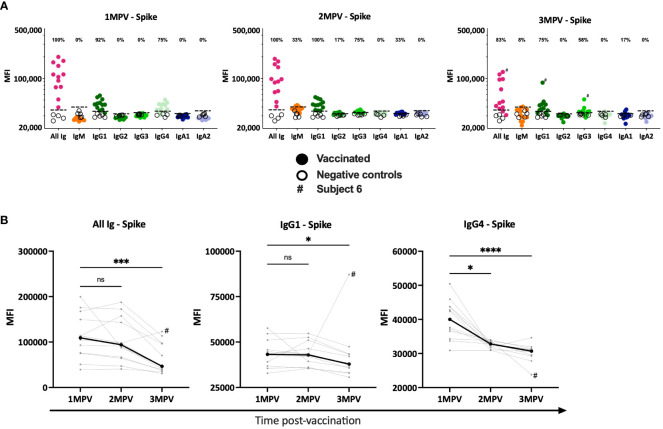
Serum antibody isotypes against SARS-CoV-2 spike detected at the different time points after Ad26/Ad5 vaccination. **(A)** The levels of total Ig, IgM, IgG1-4, IgA1 and IgA2 against spike at 1-, 2-, and 3-months after Ad26/Ad5 vaccination. The dotted black line represents the cut-off (mean + 3 standard deviation of the negative control sera). The percentages of responders with Ig isotype levels above the cut-off are shown. **(B)** Changes in the levels of spike-specific total Ig, IgG1 and IgG4 in each individuals over the three time points post-vaccination. The thicker black line represents the median. MFI, mean fluorescence intensity; MPV, months post-vaccination. ****, p<0.0001; ***, p<0.001; p<0.05; ns, p ≥0.05 by Friedman test followed by Dunn’s multiple comparisons test. # denotes subject 6 who were infected with SARS-CoV-2 prior to 3 MPV.

We then examined Ig isotypes (IgM, IgG1-4 and IgA1, 2) elicited against SARS-CoV-2 spike (**Figure 2**) and RBD (**Supplementary Figure S2**) in sera from the 12 vaccinated subjects as compared to four samples from non-vaccinated COVID-19-negative controls. Total Ig responses were tested in parallel as positive controls. Spike-specific IgG1 and IgG4 were detected in almost all vaccinated subjects at 1 MPV (75 – 100% of responders) while no spike-specific IgM, IgG2, IgG3, IgA1 and IgA2 were detected (**Figure 2A**). At 2 MPV and 3 MPV, the spike-specific IgG1 responses were still detected in 100% and 75% of responders, respectively, but IgG4 responses dropped below background as soon as 2 MPV and remained undetected at 3 MPV. Interestingly, at 2 MPV very low IgG3 responses to spike were seen in 75% of vaccinees and also in 58% at 3 MPV, including in subject 6 with a breakthrough infection (**Figure 1**). When we compared the levels of spike-specific IgG1 and IgG4 responses over time, a decrease was observed between 1 MPV and 3 MPV (median 1.2- and 1.3-fold decline for IgG1 and IgG4, respectively) (**Figure 2B**).

The results were similar for RBD-specific isotypes. IgG1 and IgG4 were detectable at 1 MPV and the levels declined over time (**Supplementary Figures S2A, B**). IgG4 dropped below background starting from 2 MPV while IgG1 remained detectable in 92 – 100% of the vaccinees. However, no RBD-specific IgG3 responses were detected at any time point (**Supplementary Figure S2A**).

### Virus neutralization activities in sera of Ad26/Ad5-vaccinated individuals

**Figure 3:**
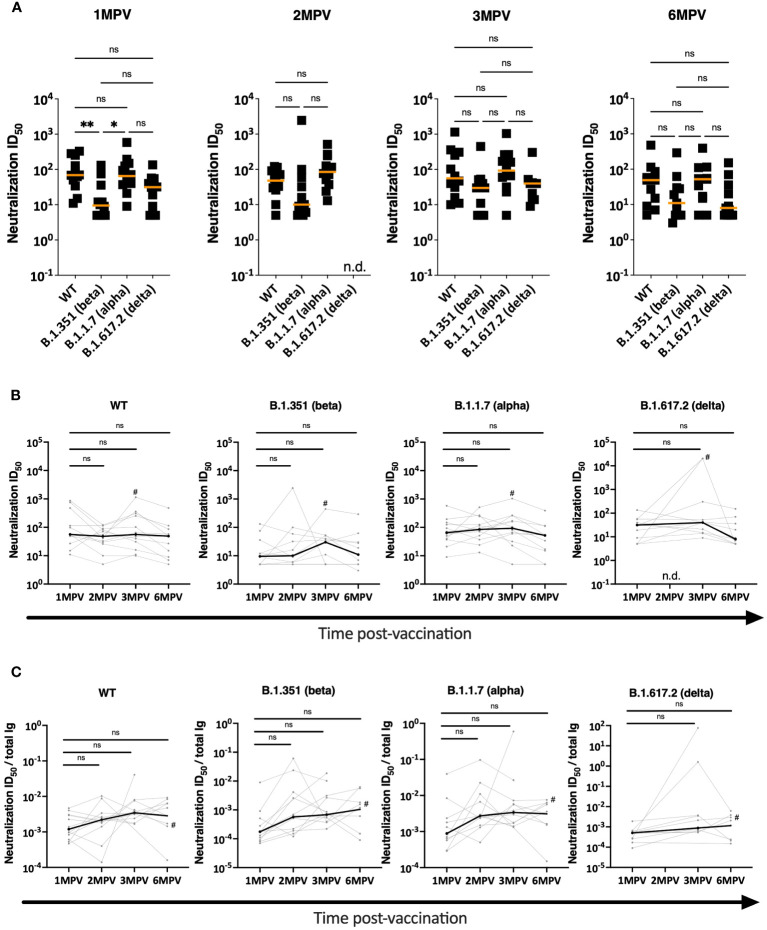
Virus neutralization activities in sera of Ad26/Ad5-vaccinated individuals. **(A)** Neutralization was measured using recombinant VSV expressing spike glycoproteins of WT, beta, alpha and delta SARS-CoV-2 strains. Serially diluted sera from Ad26/Ad5-vaccinated individuals at 1-, 2-, 3- and 6-months post-vaccination (MPV) were evaluated in the neutralization assay and the 50% inhibitory dilutions (ID_50_) were determined. The orange lines denote median values. **, p<0.01; *, p<0.05 by Kruskal-Wallis test followed by Dunn’s multiple comparisons test. **(B)** Changes in neutralization ID_50_ titers against WT, beta, alpha and delta SARS-CoV-2 strains from 1 month to later time points after vaccination. # denotes subject 6 who were infected with SARS-CoV-2 prior to 3 MPV. The thicker black lines depict median values. ns, p >0.05 by Friedman test followed by Dunn’s multiple comparisons test. **(C)** Relative potencies of neutralization were determined by calculating the neutralization activities against WT, beta, alpha and delta SARS-CoV-2 strains over total Ig levels from 1 month to later time points after vaccination. # denotes subject 6 who were infected with SARS-CoV-2 prior to 3 MPV. The thicker black lines depict median values. ns, p >0.05 by Friedman test followed by Dunn’s multiple comparisons test.

The neutralization capacities of the vaccinees’ sera were evaluated against recombinant vesicular stomatitis virus (VSV) expressing spike glycoprotein of WT (Wuhan Hu-1), beta, alpha, and delta strains (**Figure 3**). There was a trend of lower neutralizing titers against the beta and delta strains as compared to WT and alpha, similar to the pattern previously reported (16) and also seen with neutralizing activities induced by LNP-mRNA vaccines and after infection (25). The same trend was maintained from 1 MPV to 6 MPV. With a limited sample size (n = 12), a significantly lower titer was observed only against the beta vs. WT and alpha strains at 1 MPV (**Figure 3A**). When we examined the neutralization titers over time against each of the four different strains, we observed no significant decline in titers between 1 MPV and the subsequent time points (**Figure 3B**). These results indicate the stability of neutralization titers against the four variants over time in spite of declining spike-binding Ab levels during the same duration (**Figure 1**), in agreement with previously reported data (26). When we normalized the neutralization titers to the spike-binding Ig levels as ratios of ID_50_ to anti-spike total Ig AUC at 1 MPV vs. 6 MPV, a trend of increased ratios was observed that varied depending on the viral strains (from 2 to 5-fold increase) and did not reach statistical significance with this limited sample size (**Figure 3C**). Overall, the data indicate the maintenance of neutralizing potency against the early virus strains over six months post-vaccination.

### Fc-mediated binding activities of serum antibodies detected after Ad26/Ad5 vaccination declined over time

**Figure 4:**
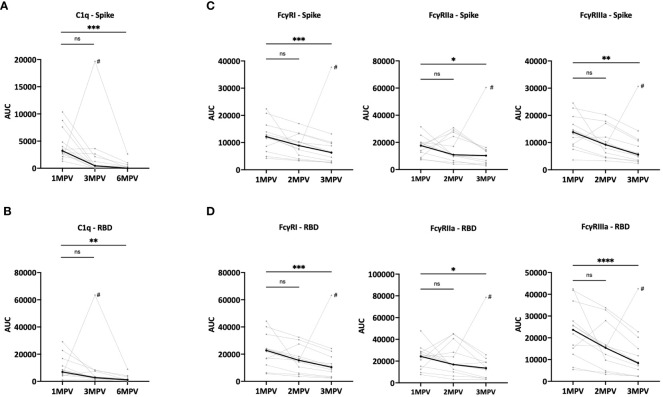
Fc-mediated binding activities of serum antibodies detectable over time after Ad26/Ad5 vaccination. **(A, B)** Changes in C1q binding activity of **(A)** spike- or **(B)** RBD-specific antibodies induced by Ad26/Ad5 vaccination from 1-month post-vaccination (MPV) to 3MPV and 6 MPV. C1q binding was measured in a multiplex bead assay in which spike- or RBD-coated beads were reacted with serially diluted serum, washed, and then treated with C1q. C1q binding was detected using a PE-conjugated anti-C1q secondary antibody. **(C, D)** Reduction in Fc receptor binding activities of **(C)** spike- or **(D)** RBD-specific Ad26/Ad5-induced antibodies from 1 MPV to 3 MPV or 6 MPV. The binding of recombinant FcγRI, FcγRIIa, and FcγRIIIa proteins to **(C)** spike- or **(D)** RBD-specific antibodies was detected in multiplex bead assays using recombinant Fc receptors with His-Tag and PE-conjugated anti-His secondary antibody. AUC, area under the curve. # denotes subject 6 who were infected with SARS-CoV-2 prior to 3MPV. The thicker black lines represents the median values. ****, p<0.0001; ***, p<0.001; **, p<0.01; *, p<0.05; ns, p ≥0.05 by Friedman test followed by Dunn’s multiple comparisons test.

In addition to virus neutralizing activities, we assessed the Fc-mediated capacities of spike- and RBD-specific antibodies in sera from the Ad26/Ad5 vaccine recipients. To this end, we measured C1q and FcγR binding using C1q and FcγRI, IIa and IIIa proteins in the multiplex bead assay (**Figure 4**, **Supplementary Figures S3**, **S4**). Titrating amounts of C1q and FcγR binding to both spike- and RBD-specific antibodies were observed, and at 1 MPV the binding levels were above background for all 12 vaccine recipients (**Supplementary Figures S3**, **S4**). To show changes in the C1q and FcγR binding activity over time, AUC was calculated from each of the titration curves.

The C1q binding levels to spike- (**Figure 4A**) and RBD-specific (**Figure 4B**) antibodies varied among samples from the 12 vaccinees but all declined between 1 MPV and 6 MPV, with median fold decreases of 221 for spike and 6.1 for RBD. At 6 MPV, very low or no C1q binding activities were detectable (**Supplementary Figures S3**, **S4**). The declines were also noted from 1 MPV and 3 MPV at 7.1-fold for spike and 2.5-fold for RBD. The magnitude of the decrease was faster than that of the total Ig levels for spike but was comparable for RBD (**Supplementary Figure S5**).

Similarly, the binding of FcγRI, IIa and IIIa receptors to spike- and RBD-specific antibodies were detected in all samples at 1 MPV, albeit at variable levels (**Figures 4C, D**, **Supplementary Figures S3**, **S4**). The levels of FcγR binding decreased significantly from 1 MPV to 3 MPV, with median fold decreases of 1.7 – 2.8 (**Figures 4C, D**). These rates of decline were not as steep as those of C1q binding (**Figures 4A, B**). The changes were slower than or consistent with the declining antibody levels observed over the same duration after vaccination (**Supplementary Figure S5**).

### Different isotype profiles of SARS-CoV-2 spike-specific serum antibodies elicited after Ad26/Ad5 vs. LNP-mRNA vaccination and after COVID-19

**Figure 5:**
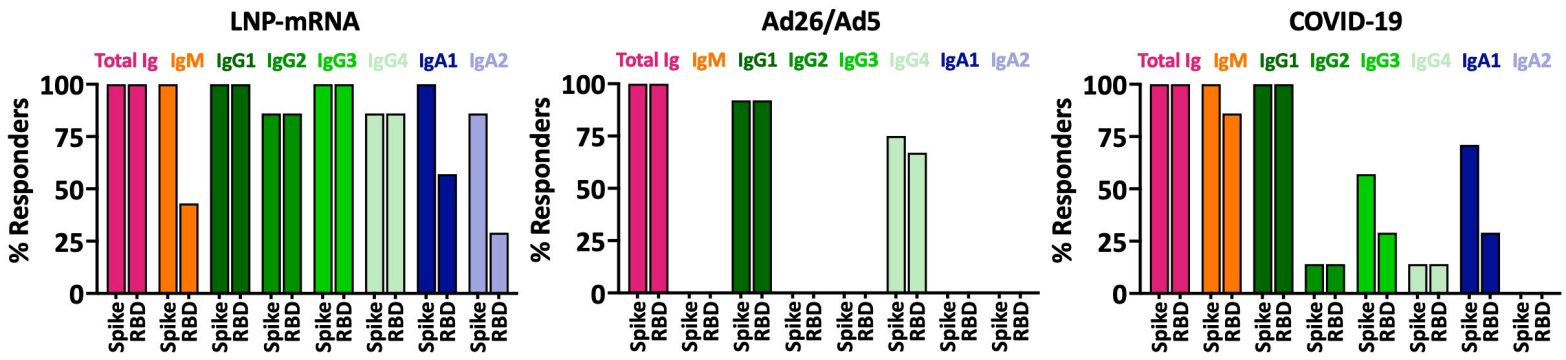
Antibody isotypes against SARS-CoV-2 spike and RBD in sera of individuals after Ad26/Ad5 vs LNP-mRNA vaccination and convalescent COVID-19 patients. Percentages of total Ig, IgM, IgG1-4, IgA1 and IgA2 responders to spike and RBD among Ad26/Ad5-vaccinated individuals at 1-month post-vaccination (1 MPV) were compared to those of LNP-mRNA-vaccinated individuals at 1 MPV and convalescent COVID-19 patients at >189 days post-symptom onset. A cutoff of mean + 3 SD of negative controls was used to determine percent responders. The Ad26/Ad5 (1 MPV) data from **Figure 2** were compared with LNP-mRNA (1 MPV) and convalescent COVID-19 (>189 days post symptom onset) data reported previously in (25, 27).

We subsequently asked whether SARS-CoV-2 spike-specific antibodies elicited by Ad26/Ad5 vaccination (1 MPV) displayed differential properties and functions as compared to the antibodies studied in our previous study from LNP-mRNA vaccinees (1 MPV after 2 doses) and from convalescent COVID-19 patients (>189 days post-symptom onset) (25, 27). First, we compared the antibody isotypes in sera of these three groups. Comparing the percentage of responders for each of the antibody isotypes tested (IgM, IgG1-4 and IgA1-2), we observed distinct profiles among the groups (**Figure 5**). Although all three groups generated IgG1 as the dominant IgG subtype, the Ad26/Ad5 vaccinees produced only IgG1 and IgG4 antibodies. In contrast, LNP-mRNA vaccination generated all four IgG subtypes as well as IgM, IgA1, and IgA2, although the frequencies varied among isotypes and for anti-spike vs. RBD antibodies. Likewise, the Ad26/Ad5 vaccinees differed from COVID-19-convalescent individuals, all of whom produced IgM and IgG1, and a fraction also had IgG2, IgG3, IgG4, and IgA1 (**Figure 5**). The same isotype profile was apparent at earlier time points after COVID-19 (<37 days post-symptom onset) (28).

### Comparable SARS-CoV-2 virus neutralization activities in sera of Ad26/Ad5-vaccinated vs. LNP-mRNA-vaccinated and COVID-19-convalescent patients

**Figure 6:**
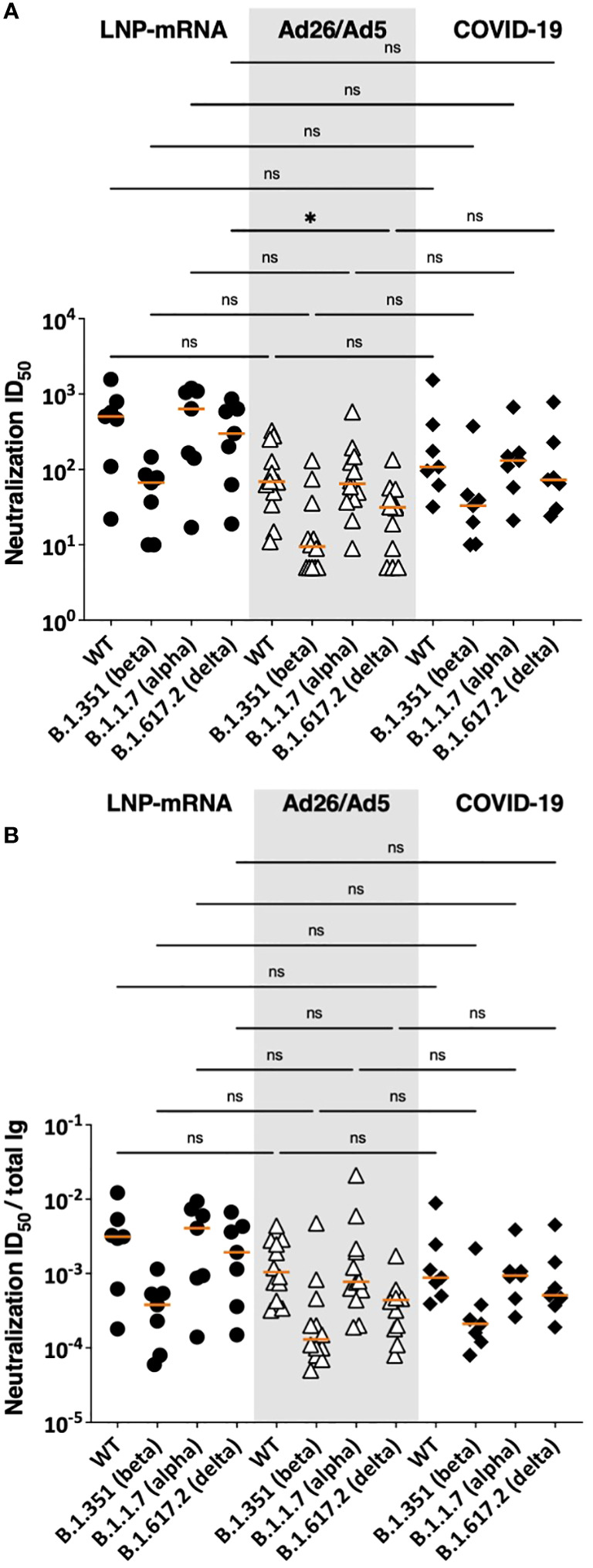
SARS-CoV-2 neutralization activities in sera of Ad26/Ad5-vaccinated vs LNP-mRNA-vaccinated and COVID-19-convalescent patients. **(A)** Serum neutralizing activities against WT, beta, alpha and delta SARS-CoV-2 strains induced by Ad26/Ad5 vaccine at 1MPV as compared to LNP-mRNA vaccine (1 MPV) or after COVID-19 at >189 days post-symptom onset (25, 27). **(B)** Relative potencies of neutralizing antibodies in the three groups as measured by the ratios of ID_50_ over total Ig levels against spike. ID_50_: 50% inhibitory dose. The orange line represents the median. *, p<0.05; ns, p ≥0.05 by Friedman test followed by Dunn’s multiple comparisons test.

Next, we assessed the functional potencies of antibodies elicited by Ad26/Ad5 vaccine vs. LNP-mRNA vaccines vs. virus infection. We compared serum neutralization ID_50_ titers against WT, alpha, beta and delta strains observed in the three groups (**Figure 6**). Except for lower neutralization titers against the delta strain in Ad26/Ad5- vs. LNP-mRNA-vaccinated individuals, the overall ID_50_ titers against all four strains were similar among the Ad26/Ad5, LNP-mRNA, and COVID-19 groups (**Figure 6A**). To account for the variable levels of spike-specific antibodies in the individual samples, we also calculated the ratios of neutralization ID_50_ over spike-specific total Ig levels and observed no significant difference (**Figure 6B**), indicating comparable neutralizing potencies induced in the three groups.

### Fc-mediated activities of SARS-CoV-2 spike and RBD-specific serum antibodies elicited after Ad26/Ad5 vaccination vs. LNP-mRNA vaccination and after recovery from COVID-19

**Figure 7:**
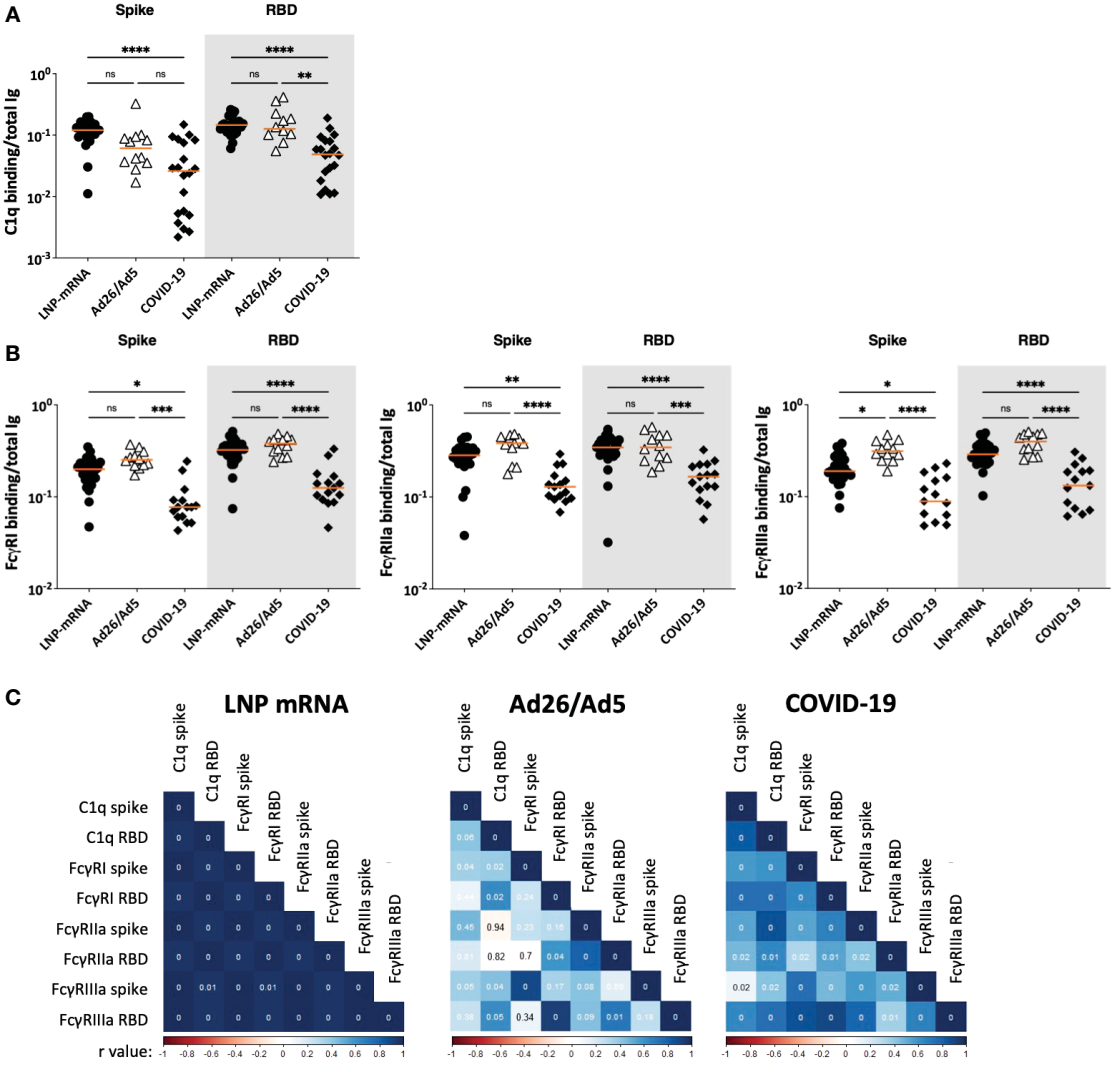
Fc-mediated activities of SARS-CoV-2 spike and RBD-specific serum antibodies elicited after Ad26/Ad5 vaccination vs LNP-mRNA vaccination and after recovery from COVID-19. **(A)** C1q-binding potencies of spike and RBD-specific antibodies elicited in the three different groups. **(B)** Potencies of spike and RBD-specific antibodies from the three groups to engage FcγRI, FcγRIIa, and FcγRIIIa receptors. The relative potencies of Fc activities were determined by calculating the ratios of C1q or Fc receptor binding levels over total Ig levels against the respective antigens. Ad26/Ad5 data (1 MPV) were compared with LNP-mRNA (1 MPV) and convalescent COVID-19 (>189 days post symptom onset) data from (25, 27). The orange line represents the median. ****, p<0.0001; ***, p<0.001; **, p<0.01; *, p<0.05; ns, p ≥0.05 by Friedman test followed by Dunn’s multiple comparisons test. **(C)** Spearman correlation matrices were generated to show how the different Fc functional activities correlated with each other in the three studied groups. The Spearman r values are color coded from red to blue. The p values are shown in the correlation boxes, with 0 indicating p<0.01.

**Figure 8:**
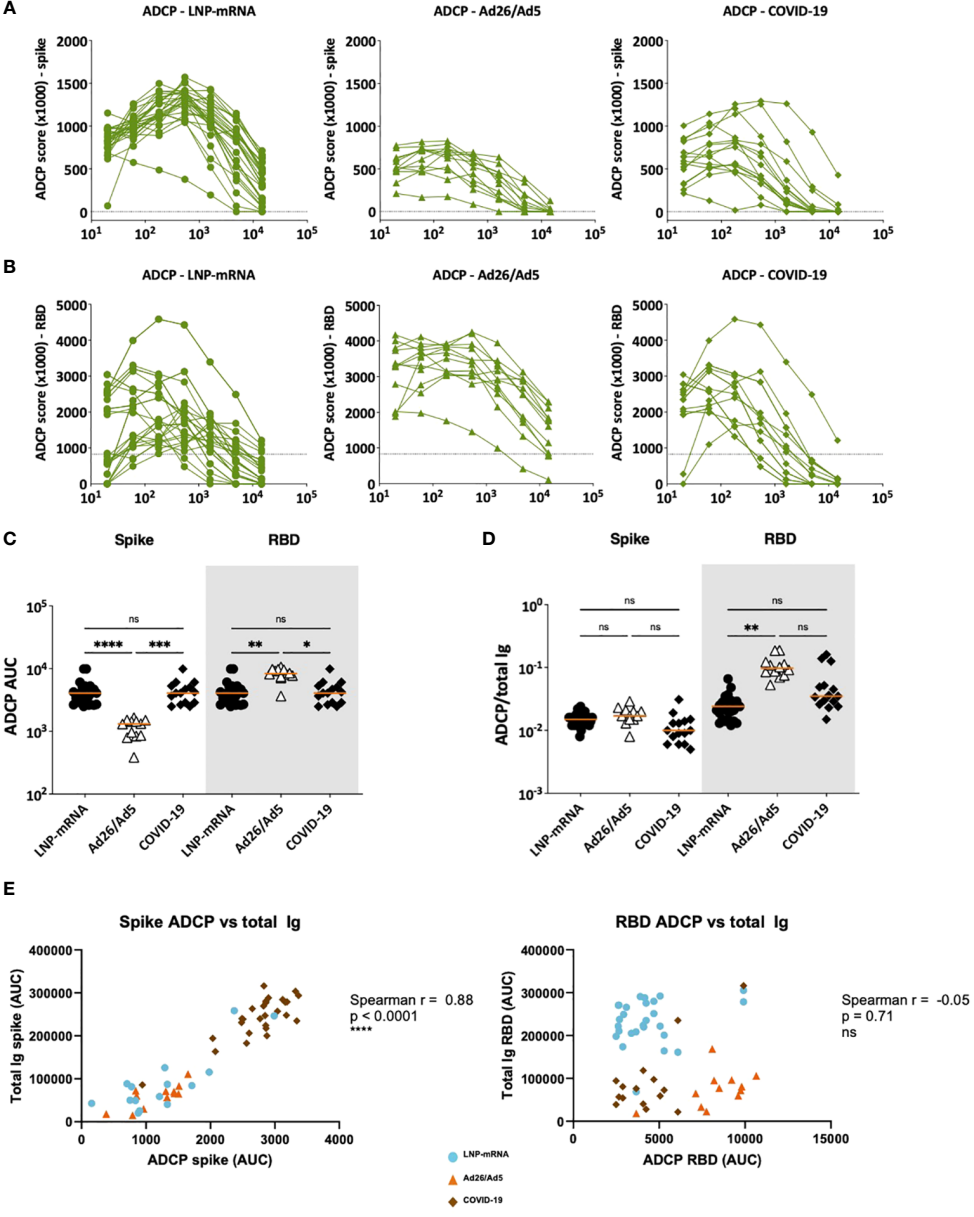
ADCP activities displayed by serum antibodies elicited after Ad26/Ad5 vaccination, LNP-mRNA vaccination, or after recovery from COVID-19. **(A)** Spike- and **(B)** RBD-specific ADCP activities in serially diluted sera of the three different groups. The dotted lines represent the cut-off above control sera. Area under the curve (AUC) was calculated from each titration curve. **(C)** ADCP AUC of spike and RBD-specific antibodies elicited in the three different groups. **(D)** Relative ADCP potencies of spike-and RBD-specific antibodies elicited in the three different groups as measured by the ratios of ADCP over total Ig levels against the respective antigens. The orange line represents the median. ****, p<0.0001; ***, p<0.001; **, p<0.01; *, p<0.05; ns, p ≥0.05 by Friedman test followed by Dunn’s multiple comparisons test. **(E)** Spearman correlations of spike-specific total Ig binding vs ADCP AUC (left) and RBD-specific total Ig binding vs ADCP AUC (right).

Finally, we compared the potencies of Fc-mediated activities mediated by spike and RBD-specific serum antibodies elicited in the Ad26/Ad5, LNP-mRNA, and COVID-19 groups (**Figures 7**, **8**). To account for the different antibody levels in the sera, we calculated the ratios of Fc activities over the level of total Ig binding the respective antigens. Comparison of C1q deposition revealed that LNP-mRNA-induced antibodies had a significantly higher potency than antibodies from COVID-19 convalescent patients (**Figure 7A**), consistent with our past findings (25). Ad26/Ad5-induced antibodies also exhibited higher potencies than COVID-19-induced antibodies, although a significant difference was observed with RBD-specific and not spike-specific antibodies (**Figure 7A**). The C1q binding potencies of antibodies elicited by Ad26/Ad5 and LNP-mRNA vaccines were comparable.

For FcγR binding, a similar pattern was observed for all three activating receptors studied (FcγRI, Iia and IIIa): the Ad26/Ad5 and LNP-mRNA groups had spike- and RBD-specific antibodies with higher binding capacities for FcγRs than the COVID-19 group (**Figure 7B**). The Ad26/Ad5-induced antibodies displayed a consistent trend of higher FcγR binding capacity than the LNP-mRNA-induced antibodies, although a significant difference was achieved only for the FcγRIIIa binding of anti-spike antibodies (**Figure 7B**). The greater Fc-mediated activity induced by vaccination vs. infection was similarly observed when we measured the FcγRIIIa intracellular signaling in a cell-based assay (**Supplementary Figure S6**).

Correlation analysis of complement and FcγR binding potencies revealed distinct patterns among the three groups (**Figure 7C**). The LNP-mRNA group showed uniformly strong correlations among C1q and FcγR binding activities of spike-and RBD-specific antibodies, whereas the COVID-19 group exhibited significant but weaker correlations. In the Ad26/Ad5 group, on the other hand, poor or no correlations were observed for most of the studied parameters.

ADCP was also measured in titrated sera from the three groups (**Figures 8A, B**). The Ad26/Ad5 had lower spike-specific ADCP and higher RBD-specific ADCP than the other two groups (**Figure 8C**). However, when the serum antibody levels were accounted for by calculating the ADCP/total Ig ratios, a trend of higher ADCP potencies was observed in the Ad26/Ad5 group vs. the other two groups, albeit a significant difference was attained only for RBD-specific ADCP between the Ad26/Ad5 and LNP-mRNA groups (**Figure 8D**). Notably, whereas the levels of spike-specific total Ig correlated with spike-specific ADCP (**Figure 8E** left panel), correlation was absent between RBD-specific total Ig and RBD-specific ADCP for all three groups (**Figure 8E** right panel), suggesting potential differences in Fc functions of antibodies targeting different regions or epitopes of spike. Altogether, the examination of Fc functions revealed the distinctive qualities of antibodies elicited by Ad26/Ad5 vaccines, LNP-mRNA vaccines, and SARS-CoV-2 infection.

## Discussion

Our study demonstrated that SARS-CoV-2 spike-specific serum antibody responses elicited by Ad26/Ad5 vaccines were dominated by the IgG1 subtype. IgG4 antibodies were also detected albeit at low levels and transiently at 1 MPV only, while other IgG subtypes and Ig isotypes were undetectable, indicating the narrow Ig isotype profile as compared to those elicited by LNP-mRNA vaccines or natural infection. Similar to previous observations (15), the antibody responses declined significantly over 6 MPV. However, serum neutralization titers against the original Wuhan strain and other early variants of concern were stable over time. When changes in spike-binding antibody levels were accounted for, a trend of increasing neutralizing potency was apparent, which suggested continuing antibody maturation over 6 MPV, similar to the antibody evolution observed in convalescent COVID-19 patients (29, 30). We also noted that the neutralizing titers and breadth against the early strains of SARS-CoV-2 were comparable for the Ad26/Ad5 vaccine, LNP-mRNA vaccine and convalescent COVID-19 groups. In contrast, the complement binding, FcγR binding, and ADCP potencies of SARS-CoV-2-specific antibodies induced by the Ad26/Ad5 and LNP-mRNA vaccines were significantly higher or trended higher than those induced by virus infection, independent of binding Ig levels. Distinct patterns in the Fc function correlations further pointed to the differences among the three groups, although the constellation of Fc functions for optimal protection against disease is yet unknown (29, 30).

The reasons for high Fc functional capacities generated by the Ad26/Ad5 vaccines are not understood. A previous study reported the FcγR-binding activities to be more robust in the recipients of LNP-mRNA vaccines (Pfizer/BNT162b2 and Moderna/mRNA-1273) vs. vectored vaccines (AstraZeneca/ChadOx1/AZD1222 and Janssen/Ad26.COV2.S), but the varying Ig binding levels were not considered (31). When normalized to the antibody levels, Fc mediated activities such as ADCP and antibody-dependent complement deposition (ADCD) were found higher in the vectored vaccine groups than the LNP-mRNA vaccine groups (31). A study comparing the CoronaVac (aka Sinovac, whole inactivated virus vaccine) and Pfizer/BioNTech mRNA vaccines showed strong differences in peak antibody binding titers and Fc-effector functions across the 2 vaccine platforms, which both waned with time (32). Moreover, CoronaVac-induced Fc-effector functions demonstrated a steep and rapid decline to undetectable levels while binding to IgG was still present, suggesting that the binding antibody level does not directly translate to the level of antibody effector function. In another report, compromised FcγR binding activities against different spike variants of concern were observed in convalescent COVID-19 patients as compared with LNP-mRNA vaccine recipients, and the differences were ascribed to lower antibody quantities present in the convalescent samples (33). In this study the inferior FcγR-binding potencies of antibodies from convalescent patients were evident even after the levels of spike- and RBD-binding antibodies were taken into account.

The Ig isotype profile in the Ad26/Ad5 vaccinated subjects was distinct from those of LNP-mRNA vaccines and convalescent COVID-19 groups, but this is not likely to explain the high FcγR binding activities seen in the Ad26/Ad5 vaccine group. Of the four human IgG subclasses, IgG3 has the highest affinity for FcγRIIIA and the most potent for engaging complement (34–36). IgG3 was induced by the LNP-mRNA vaccines and SARS-CoV-2 infection, but not by the Ad26/Ad5 vaccines. IgG4, which was detected in the Ad26/Ad5 vaccinees, does not bind C1q and has low affinity for the tested FcγR (34–36). The IgG1 responses were similarly dominant in all three groups; however, the glycosylation differences may contribute to higher Fc potencies in the Ad26/Ad5 group. The addition or removal of fucose, galactose and bisecting N-acetylglycosamine to the IgG Fc glycan core create distinct arrays of Fc glycoforms that modulate Fc affinity and effector functions (37–41). Further investigations are needed to determine the relative Fc glycan contents of SARS-CoV-2-specific antibodies from the three studied groups.

Our data point to the ability of adenoviral vaccine platforms to evoke strong Fc-mediated activities, but the potential role of robust Fc effector functions should be considered not only for protection but also in the development of immunopathogenesis. Spike- and RBD-specific antibody-mediated complement activation and phagocytosis have been linked to viral control in the bronchoalveolar fluid (42). Moreover, spike-specific phagocytic and complement binding activity were enriched in convalescents vs. deceased patients (43). In the context of SIV, the protective efficacy of adenovirus/protein vaccines against virus challenges in rhesus monkeys correlated with Fc-mediated activities such as ADCC, ADCP and ADCD (44). However, antiviral antibodies can also promote viral infection of host cells by exploiting the phagocytic FcγR pathway to result in antibody-dependent enhancement (ADE) as observed in the context of flaviviruses and particularly dengue virus (45). Nonetheless, there is no evidence for ADE in SARS-CoV-2 infection and vaccination (21). Very rare thrombosis and thromobocytopenia have been reported in previously healthy young adults after receiving the adenovirus-vectored AstraZeneca and Janssen vaccines (46). Antibodies to platelet factor 4 (PF4) from patients with these vaccine-induced thrombotic events form immune complexes and activate platelets through crosslinking of FcγRIIA, the only FcγR expressed on human platelets (47). Our study demonstrated that Ad26/Ad5 vaccines elicited spike- and RBD-specific Abs with greater binding potencies to FcγRs including FcγRIIA. However, it is not known whether the Ad26/Ad5 vaccines and other adenovirus-vectored vaccines may also influence the FcγR binding properties of antibodies against bystander antigens including PF4, and further investigation is required to examine this possibility.

The experiments presented here were limited by several factors. First, the sample size was small for each of the three studied groups and the samples were not from a single population: the Ad26/Ad5 specimens were from Argentina while the LNP-mRNA and COVID-19 groups were residents of the USA. Secondly, the binding and functional antibody assays were restricted to the original Wuhan strain or early SARS-CoV-2 alpha, beta and delta strains, although the samples studied here were collected during the early pandemic (02/22/21 to 07/19/21), when the relevant dominant variant was delta, prior to the rise of the omicron variant and its subvariants. Thirdly, the presence of antibodies against the other circulating human coronaviruses was not evaluated. SARS-CoV-2-specific antibodies display poor cross-neutralization capacities against the previously endemic human coronaviruses, but cross-reactive non-neutralizing antibodies capable of certain Fc functions have been reported (48). Lastly, the evaluation of Fc activities was limited to C1q and FcγR binding in multiplex bead assays and ADCP measurements with antigen-coated beads and THP-1 effector cells. The capacity of antibodies to exert Fc effector functions that lead to the destruction of virus or infected cells by lysis or phagocytosis were not examined.

## Methods

### Human specimens

Sera were collected from 12 Sputnik V Ad26/Ad5-vaccinated individuals recruited for this study at the Administración Nacional de Laboratorios e Institutos de Salud “Dr. Carlos Malbrán” (ANLIS MALBRAN) in Buenos Aires, Argentina (A.E.V, A.E, C.P.). Volunteers received their first dose either on 29 December or 31 December 2020, and their second dose either on 20 January 2021 or 2 February 2021. Data presented in this study were from sera collected at 1, 2, 3 and 6 months post-second dose. Serum samples were obtained from human volunteers of both sexes and de-identified prior to use in experiments. Sex was not considered as a biological variable in this study. Serum collection at ANLIS MALBRAN was approved by the Research Ethics Committee of its Unidad Operativa Centro de Contención Biológica (UOCCB) on 9 February 2021. Written informed consent was obtained as per institutional policy.

For comparison, a total of 27 specimens from the recipients of Moderna mRNA-1273 or Pfizer/BioNtech BNT162b2 LNP-mRNA vaccines (1 MPV after 2 doses), 15 specimens from convalescent COVID-19 patients (>189 days post-symptom onset), and four specimens from contemporaneous non-vaccinated COVID-19-negative subjects were also evaluated. Eighteen of these specimens were obtained from volunteers enrolled in IRB-approved protocols at the Icahn School of Medicine at Mount Sinai (IRB#17-00060, IRB#19-01243) and the James J. Peter Veterans Affairs Medical Center (IRB#BAN-1604): seven [RN#1, RN#4 and RV#1-5] after LNP-mRNA immunization; seven [RP#2-5, 7, 12, 13] after infection; and four negative controls. Eight additional convalescent plasma samples (CVAP samples) were obtained from 199-243 days after symptom onset under the JJPVAMC Quality Improvement project “Evaluation of the clinical significance of two COVID-19 serologic assays”. Twenty post-LNP mRNA vaccination plasma samples were also collected from participants in the longitudinal observational PARIS (Protection Associated with Rapid Immunity to SARS-CoV-2, IRB#20-03374) study. All participants signed written consent forms prior to sample and data collection. All participants provided permission for sample banking and sharing. All samples were heat-inactivated before use.

### Recombinant proteins

SARS-CoV-2 spike and RBD proteins were produced as described previously (49, 50). S1 (amino acids 16-685), S2 (amino acids 686-1213), and nucleoprotein (amino acids 1-419) antigens were purchased from ProSci Inc, CA (#97-087, #97-079 and #11-184, respectively). All antigens were of the parental SARS-CoV-2 Wuhan-Hu-1 or WA1 strain.

### Multiplex bead Ab binding assay

Measurement of total Ig and Ig isotypes to SARS-CoV-2 antigen-coupled beads was performed as described (25, 27, 28). The quantification was based on median fluorescent intensity (MFI) values at the designated sample dilutions. For total Ig responses, specimens were diluted 10-fold from 1:100 to 1:100,000, reacted with antigen-coated beads, and treated sequentially with biotinylated anti-human total Ig antibodies and PE-streptavidin. The isotyping assays were performed at a single 1:200 dilution using human Ig isotype or subclasses antibodies and the MFI values were shown.

For the C1q assay, beads with spike-Ab or RBD-Ab complexes were incubated with C1q Component from Human Serum (Sigma, #C1740) for 1 hour at room temperature, followed by an anti-C1q-PE antibody (Santa Cruz, #sc-53544 PE) for 30 minutes at room temperature (25, 27).

For the FcγR assays, beads with spike-Ab or RBD-Ab complexes were incubated with His-tagged recombinant FcγRI/CD64 (R&D Systems™, #1257FC050), FcγRIIa/CD32a (R&D Systems™, #1330CD050/CF) or FcγRIIIa/CD16a (R&D Systems™, #4325FC050) proteins for 1 hour at room temperature, followed by an anti-His-PE antibody (R&D Systems™, #IC050P) for 30 minutes at room temperature. FcγRIIIa signaling was also assessed using a reporter cell co-culture system according to the protocol published previously (51).

The relative levels of binding were obtained as MFI, from which titration curves were plotted and areas-under the curves (AUC) were calculated.

### Recombinant spike-VSV neutralization assay

This assay used 293T-hACE2-TMPRSS2 target cells which were seeded at a density of 4 × 10^4^ cells per well in flat-bottom 96-well plates (Fisher Scientific, #08-772-3) after coating with collagen (Millipore, #08-115). The cells were incubated at 37°C/5% CO_2_ overnight (~20 hours). On the following day, recombinant VSV virions expressing SARS-CoV Spike and a GFP reporter were pre-incubated with 4-fold serially diluted serum (starting from 1:5 to 1:20,480) in DMEM with 10%FBS and 1%PenStrep for a minimum of 10 minutes at room temperature, and then added to the target cells. At 13 hours post-infection, GFP counts were acquired by the Celigo imaging cytometer (Nexcelom Biosciences, version 4.1.3.0). Each assay was performed in triplicates.

To calculate ID_50_, GFP counts from “no serum” conditions were set to 100%; GFP counts of each serum-treated condition were normalized to no serum control well. Inhibition curves were generated using Prism 9.1.2 (225) (GraphPad Software) with “log (inhibitor) vs. normalized response-variable slope” settings.

### Antibody-dependent cellular phagocytosis

Assays to measure spike-specific ADCP were performed using a protocol reported previously (25, 27). Briefly, FluoSpheres carboxylate-modified yellow-green fluorescent microspheres (Thermo Fisher, #F8823) were coupled with SARS-CoV-2 spike or RBD proteins using the xMAP Antibody Coupling Kit (5 µg protein/~36.4x10^9^ beads, Luminex #40-50016). Spike-conjugated microspheres were incubated with diluted plasma for 2 hours at 37°C in the dark. After washing and centrifugation (2,000 g, 10 min), the beads (~3x10^8^ beads, 10 µL/well) were incubated with THP-1 cells (0.25x10^5^ cells, 200 µL/well) for 16 hours. The samples were analyzed on an Attune NxT flow cytometer (Thermo Fisher, #A24858). Data analysis was performed using FCS Express 7 Research Edition (*De Novo* Software). ADCP scores were calculated as follows: (% microsphere positive cells) x (geometric mean fluorescent intensity of the microsphere positive cells)/1000.

### Statistics

Statistical analyses were performed as designated in the figure legends using GraphPad Prism 9 (GraphPad Software, San Diego, CA). Correlation matrices were generated using R version 4.1.0 (The R Foundation for Statistical Computing) and corrplot package.

## Data availability statement

The original contributions presented in the study are included in the article/**Supplementary Material**. Further inquiries can be directed to the corresponding author.

## Ethics statement

The studies involving humans were approved by Research Ethics Committee of its Unidad Operativa Centro de Contención Biológica (UOCCB); Icahn School of Medicine at Mount Sinai Institutional Research Board; James J. Peter Veterans Affairs Medical Center Institutional Research Board. The studies were conducted in accordance with the local legislation and institutional requirements. The participants provided their written informed consent to participate in this study.

## Author contributions

JK: Data curation, Formal analysis, Investigation, Methodology, Visualization, Writing – original draft, Writing – review & editing. SK: Data curation, Formal analysis, Investigation, Methodology, Writing – review & editing. JB: Data curation, Project administration, Resources, Writing – review & editing. RE-G: Resources, Writing – review & editing. RA: Data curation, Formal analysis, Investigation, Methodology, Writing – review & editing. PR: Data curation, Methodology, Writing – review & editing. FA: Resources, Writing – review & editing. CG: Data curation, Resources, Writing – review & editing. GK: Data curation, Resources, Writing – review & editing. VS: Data curation, Funding acquisition, Resources, Supervision, Writing – review & editing. AE: Data curation, Resources, Writing – review & editing. CP: Data curation, Resources, Supervision, Writing – review & editing. CU: Formal analysis, Methodology, Supervision, Writing – review & editing. BL: Data curation, Funding acquisition, Methodology, Resources, Supervision, Writing – review & editing. CH: Conceptualization, Funding acquisition, Project administration, Resources, Supervision, Writing – original draft, Writing – review & editing.
