# Supplementary material for: Heterologous Ad26/Ad5 adenovirus-vectored vaccines elicited SARS-CoV-2-specific antibody responses with potent Fc activities
Source: Front Immunol. 2024 May 8;15:1382619. doi: 10.3389/fimmu.2024.1382619 (PMC11109367; doi:10.3389/fimmu.2024.1382619)
Supplement: Supplementary file 1 [file DataSheet_1.pdf]

Figure S1

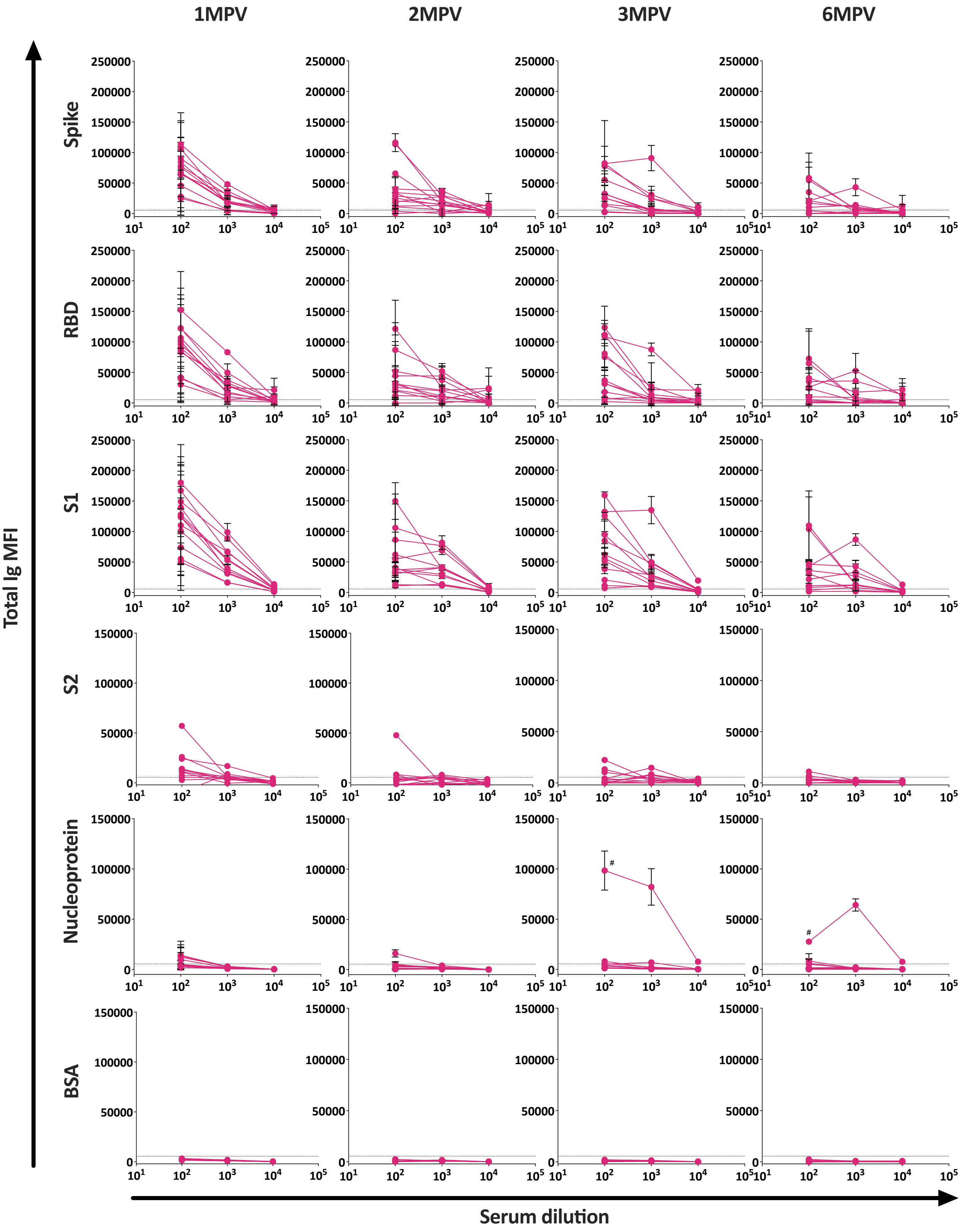

1MPV
2MPV
3MPV
6MPV
Spike
RBD
S1
S2
Nucleoprotein
BSA
Total Ig MFI
Serum dilution
#

Figure S1

Fig S1. Total Ig antibody responses to SARS-CoV-2 over 4 time points after immunization with the Ad26/Ad5 vaccines.

Sera from 12 Ad26/Ad5-vaccinated individuals were collected at 1-, 2-, 3- and 6-months post-vaccination (MPV), serially diluted, and examined for total Ig levels against spike, RBD, S1, S2, nucleoprotein and BSA control in a multiplex bead assay.

MFI: mean fluorescence intensity. The dotted line represents the cut-off based on BSA control. # denotes subject 6 who were infected with SARS-CoV-2 prior to 3MPV. Area under the curve (AUC) values were calculated from these titration curves and shown in Figure 1.

Figure S2

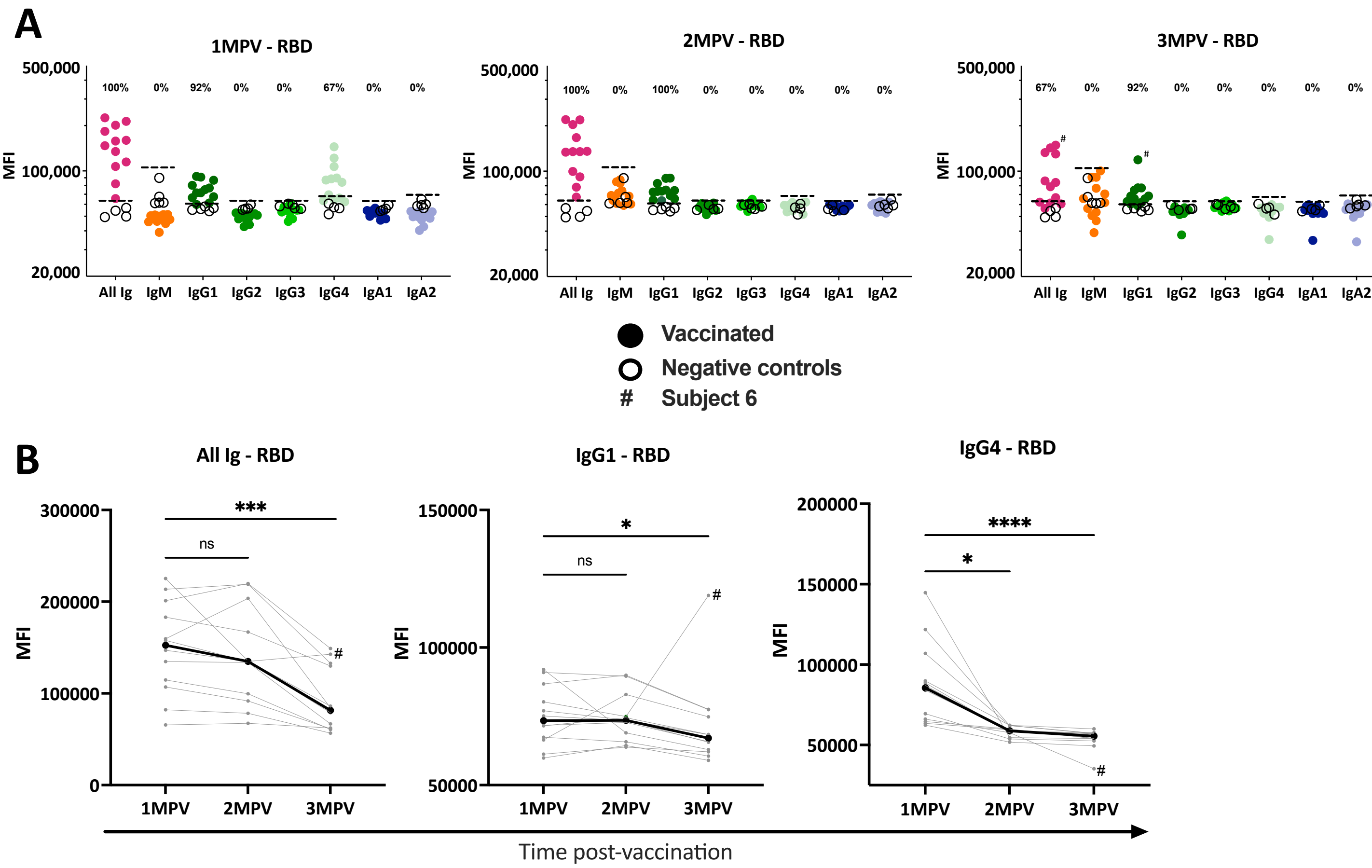

Fig S2. Serum antibody isotypes against SARS-CoV-2 RBD detected at the different time points after Ad26/Ad5 vaccination.

(A) The levels of total Ig, IgM, IgG1-4, IgA1 and IgA2 against RBD at 1-, 2-, and 3-months after Ad26/Ad5 vaccination. The dotted black line represents the cut-off (mean + 3 standard deviation of the negative control samples). The percentages of responders with Ig isotype levels above the cut-off are shown.

(B) Changes in the levels of RBD-specific total Ig, IgG1 and IgG4 in each individuals over the three time points post-vaccination. The thicker black line represents the median.

MFI: mean fluorescence intensity. MPV: months post-vaccination. ****, $p < 0.0001$; ***, $p < 0.001$; *, $p < 0.05$; ns, $p \geq 0.05$ by Friedman test followed by Dunn's multiple comparisons test.

# denotes subject 6 who were infected with SARS-CoV-2 prior to 3 MPV.

Figure S3

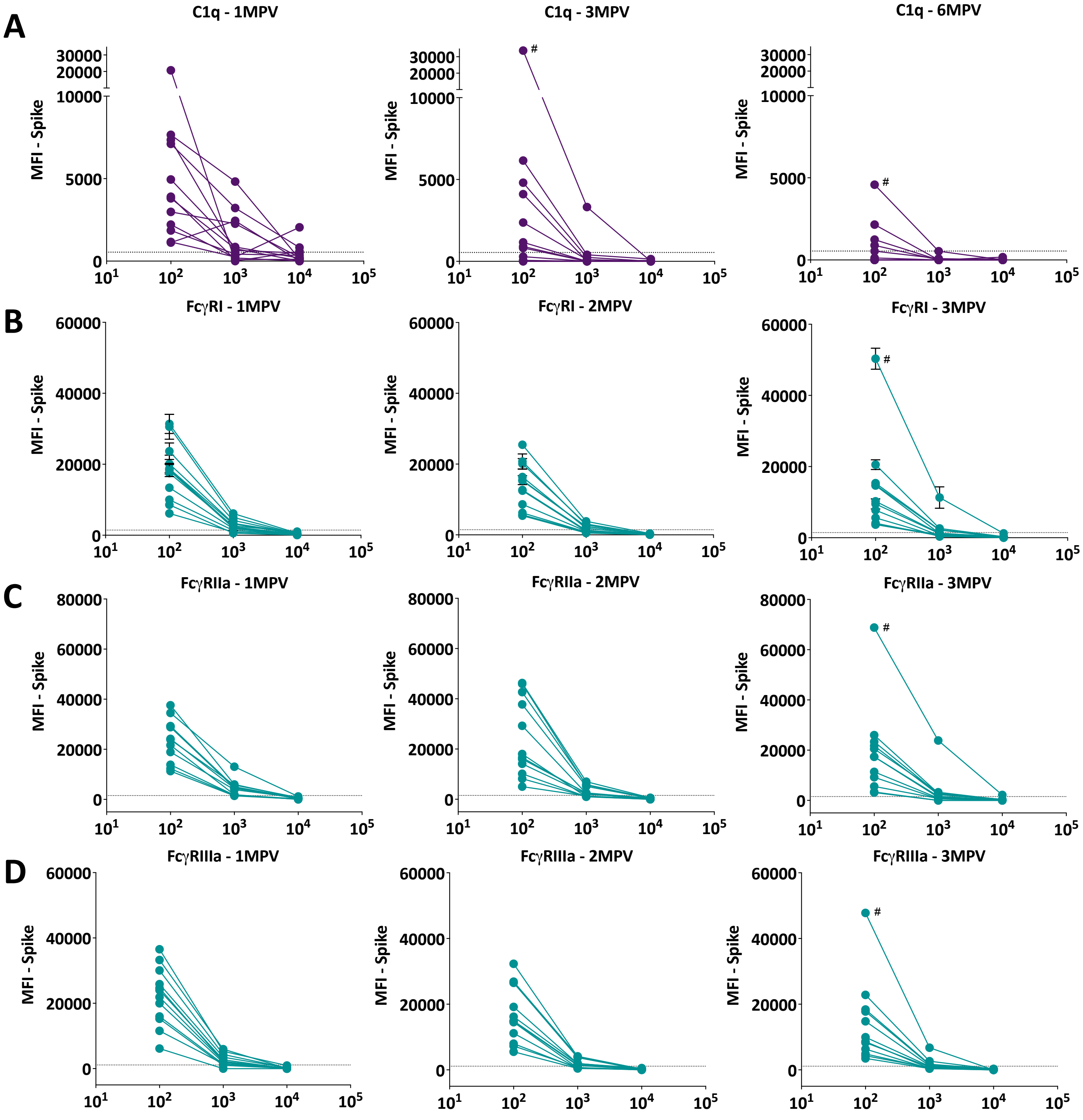
A
C1q - 1MPV
C1q - 3MPV
C1q - 6MPV
MFI - Spike
30000
20000
10000
5000
0
#
B
FcγRI - 1MPV
FcγRI - 2MPV
FcγRI - 3MPV
60000
40000
20000
C
FcγRIIa - 1MPV
FcγRIIa - 2MPV
FcγRIIa - 3MPV
80000
D
FcγRIIIa - 1MPV
FcγRIIIa - 2MPV
FcγRIIIa - 3MPV
10^1 10^2 10^3 10^4 10^5

Figure S3

Fig S3. Fc-mediated activities of spike-specific serum antibodies detected over time after Ad26/Ad5 vaccination.
(A) C1q binding to spike-specific antibodies in serially diluted sera of Ad26/Ad5-vaccinated individuals at 1 MPV, 3 MPV, and 6 MPV.
(B-D) Fc receptor binding to spike-specific antibodies in serially diluted sera of Ad26/Ad5-vaccinated individuals at 1 MPV, 2 MPV, and 3 MPV. Recombinant His6-tagged FcγRI, FcγRIIa, and FcγRIIIa proteins were used.
The dotted lines represent the cut-off above control sera. Area under the curve (AUC) was calculated from each titration curve and presented in Figure 4. MPV: month(s) post-vaccination; MFI: mean fluorescence intensity. # denotes subject 6 who were infected with SARS-CoV-2 prior to 3 MPV.

Figure S4

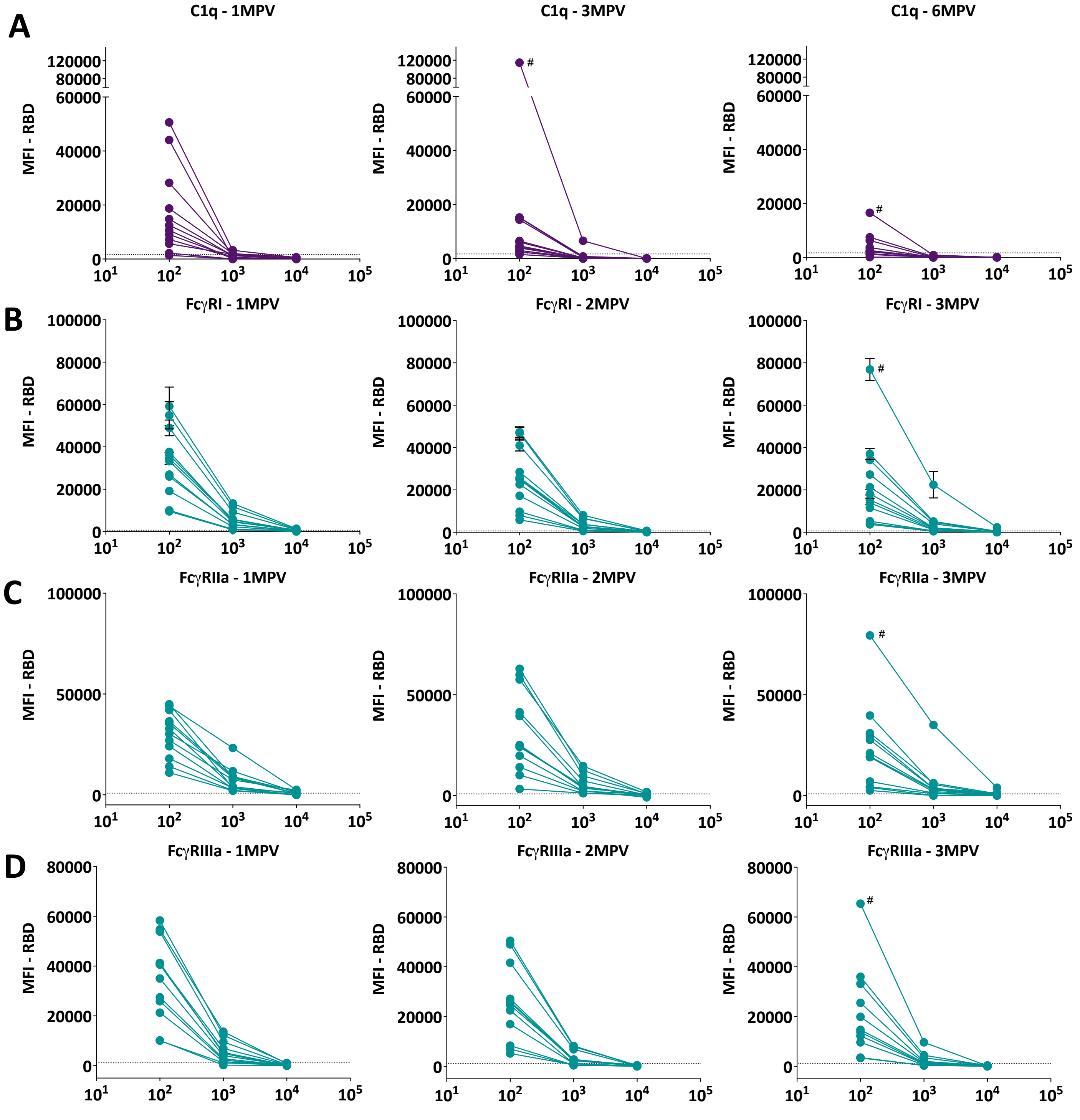
A
C1q - 1MPV
C1q - 3MPV
C1q - 6MPV
B
FcγRI - 1MPV
FcγRI - 2MPV
FcγRI - 3MPV
C
FcγRIIa - 1MPV
FcγRIIa - 2MPV
FcγRIIa - 3MPV
D
FcγRIIIa - 1MPV
FcγRIIIa - 2MPV
FcγRIIIa - 3MPV
MFI - RBD
#

Figure S4

Fig S4. Fc-mediated activities of RBD-specific serum antibodies detected over time after Ad26/Ad5 vaccination.

(A) C1q binding to RBD-specific antibodies in serially diluted sera of Ad26/Ad5-vaccinated individuals at 1 MPV, 3 MPV, and 6 MPV.

(B-D) Fc receptor binding to RBD-specific antibodies in serially diluted sera of Ad26/Ad5-vaccinated individuals at 1 MPV, 2 MPV, and 3 MPV. Recombinant His6-tagged FcγRI, FcγRIIa, and FcγRIIIa proteins were used.

The dotted lines represent the cut-off above control sera. Area under the curve (AUC) was calculated from each titration curve and presented in Figure 4. MPV: month(s) post-vaccination; MFI: mean fluorescence intensity. # denotes subject 6 who were infected with SARS-CoV-2 prior to 3 MPV.

Figure S5

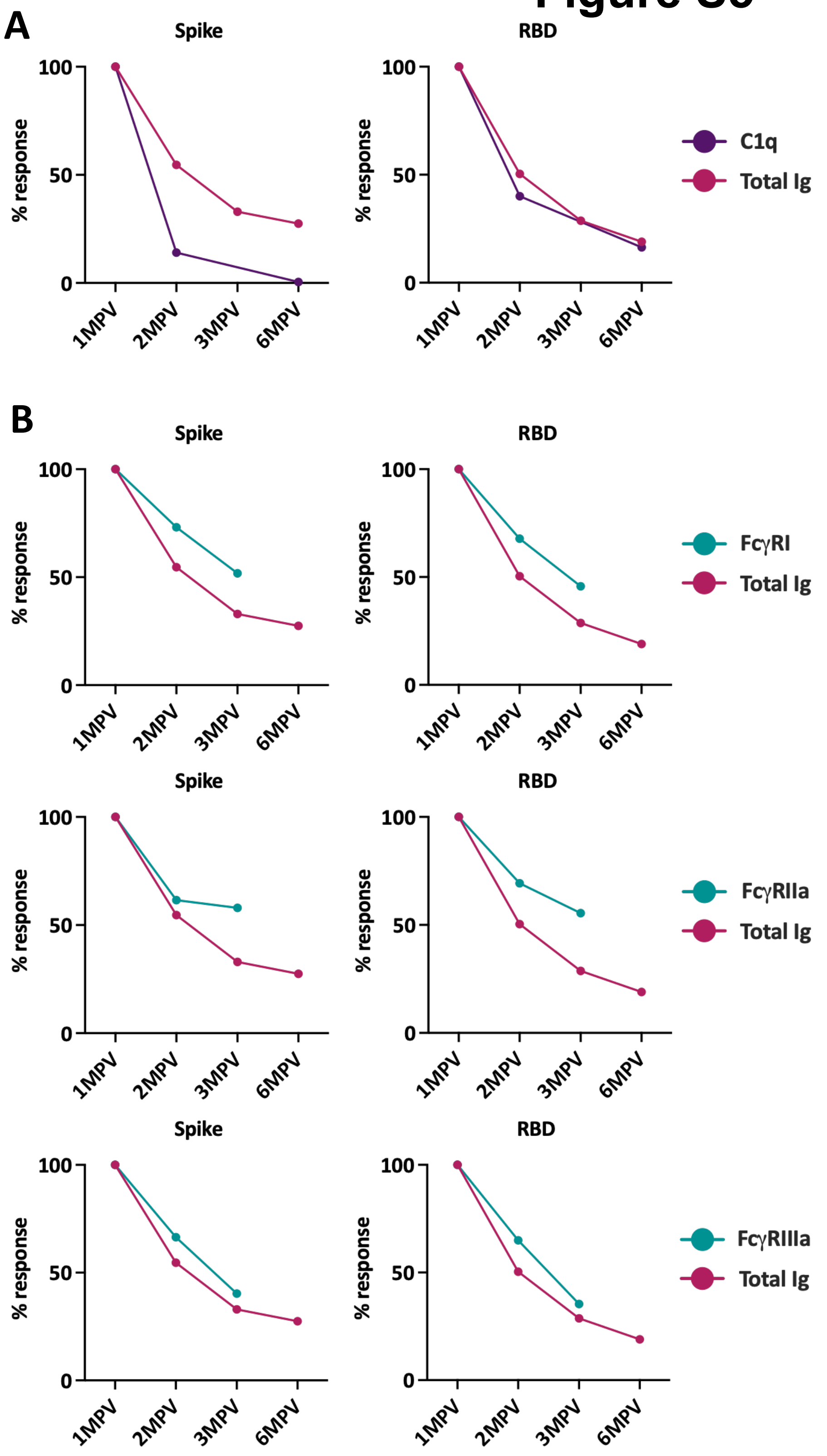

Fig S5. Relative decline rates of spike- and RBD-specific antibody Fc-mediated activities as compared to the levels of total Ig binding to the respective antigens over time after Ad26/Ad5 vaccination.

(A) Decline rates of C1q binding activities (purple) vs total Ig levels (pink).

(B) Decline rates of FcγRI, IIa and IIIa binding activities (blue) vs total Ig levels (pink).

For comparison, median values from 4 time points (1, 2, 3 and 6MPV) shown in Fig 1 and Fig 4 are normalized to the first time point (1MPV set to 100%).

Figure S6

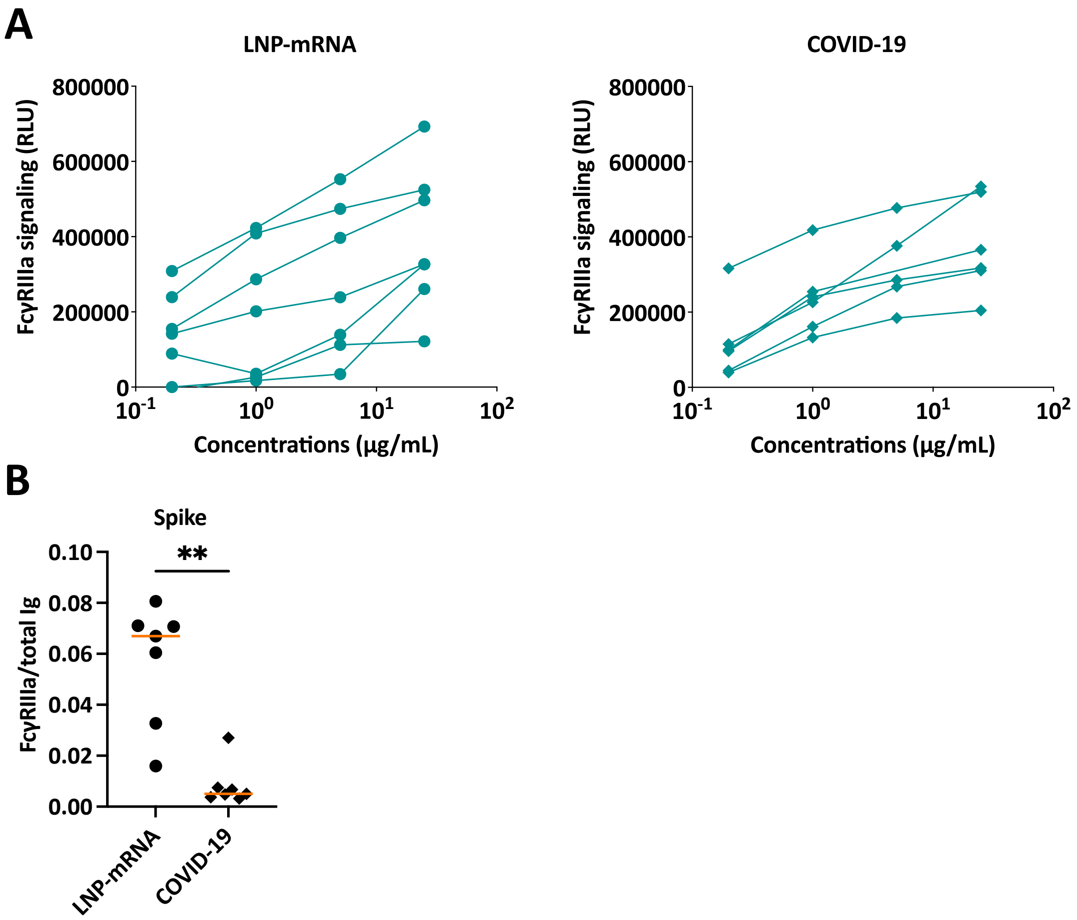

Fig S6. FcγRIIIa signaling capacity of spike-specific antibodies elicited by LNP-mRNA vaccination vs after recovery from COVID-19.

(A) The capacity of spike-specific antibodies to induce FcγRIIIa signal activation was measured as in (53). Spike-expressing transfected 293T cells were treated with serially diluted purified IgG from LNP-mRNA-vaccinated individuals (1 MPV) and COVID-19 convalescent patients (>189 days post symptom onset) and then incubated with a luciferase-inducible FcγRIIIa reporter cell line. Dose-dependent signal activation was measured by luciferase activity. RLU: Relative Light Units.

(B) The relative capacity of spike-specific antibodies to induce FcγRIIIa signaling was measured by calculating the ratios of FcγRIIIa signaling levels over spike-specific total Ig binding levels in the respective samples from LNP-mRNA-vaccinated individuals (1 MPV) and COVID-19 convalescent patients (>189 days post symptom onset). The orange line represents the median. **, $p < 0.01$ by Mann-Whitney test.
